# Integration multiplanarer Bildgebung verkürzt die Dauer einer umfassenden intraoperativen transösophagealen echokardiographischen Untersuchung

**DOI:** 10.1007/s00101-025-01631-5

**Published:** 2026-01-23

**Authors:** Waseem Zakaria Aziz Zakhary, Klara Treß, Anna Flo Forner, Jörg Ender, Massimiliano Meineri

**Affiliations:** Abteilung für Anästhesiologie und Intensivmedizin, Herzzentrum Leipzig, Struempellstrasse 39, 04289 Leipzig, Deutschland

**Keywords:** Herzchirurgie, Intraoperative Bidgebung, 3D Echokardiographie, Multiplanare Bildgebung, Cardiac surgery, Intraoperative imaging, 3D echocardiography, Multiplanar imaging

## Abstract

**Hintergrund:**

Eine umfassende transösophageale echokardiographische Untersuchung (TEE) ist bei herzchirurgischen Eingriffen heutzutage gelebte Praxis. Die Einführung der 3D-Technologie erlaubt eine simultane multiplanare Darstellung von 2D-Schnitten (X-Plane).

**Fragestellung:**

Wird durch die Integration der Multiplan-Bildgebung die Dauer einer umfassenden TEE-Untersuchung verkürzt, ohne die Genauigkeit der routinemäßigen zweidimensionalen anatomischen Linearmessungen zu beeinträchtigen?

**Material und Methoden:**

In einer prospektiven randomisierten Vergleichsstudie erhielten Patienten, die sich einer elektiven herzchirurgischen Operation unterzogen, eine umfassende TEE-Untersuchung (Philips CX 50 mit X‑72T Sonde). Es wurden 2 Gruppen gebildet: In der Gruppe „Routineprotokoll“ (RP) erfolgte der bisher übliche, abteilungsinterne Untersuchungsgang; in der Gruppe „Studienprotokoll“ (SP) wurden X‑Plane Schnittbilder in das RP integriert. Die Untersuchungen wurden von 2 erfahrenen Untersuchern durchgeführt. Ausgewählte Messungen wurden im Anschluss offline durchgeführt. Zum Vergleich mit den Messungen aus dem RP und SP wurden als Goldstandard die Messungen basierend auf einem Full-Volume-3D-Datensatz herangezogen.

**Ergebnisse:**

Die benötigte Untersuchungszeit war in der SP-Gruppe signifikant kürzer als in der RP-Gruppe (SP: 481 ± 60 s; RP 595 ± 60 s; *p* < 0,001). Bei ausgewählten Messungen gab es keinen signifikanten Unterschied.

**Schlussfolgerung:**

Die Integration multiplanarer Bildgebung in einen umfassenden TEE-Untersuchungsablauf verkürzt die Untersuchungszeit, ohne die Genauigkeit linearer Messungen zu beeinträchtigen.

**Graphic abstract:**

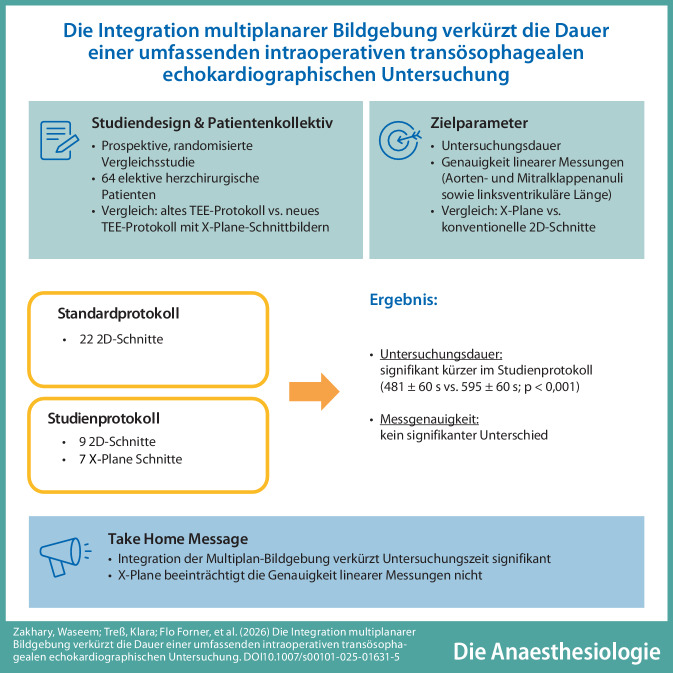

## Abstract (en)

**Background:**

A comprehensive transesophageal echocardiography (TEE) examination is nowadays common practice in cardiac surgical procedures. The introduction of 3D technology enables a simultaneous multiplane display of 2D views (X-plane) which can shorten the duration of the TEE examination; however, X‑plane imaging results in a decrease in temporal resolution which can affect the accuracy of routine anatomical linear measurements. The 3D-TEE imaging enables multiplanar reconstruction of 3D datasets and to freely position perpendicular 2D planes to measure anatomical structures more accurately, which has been shown to strongly correlate to computed tomography and magnetic resonance imaging.

**Objective:**

Does the integration of multiplane imaging shorten the time of a comprehensive TEE examination without affecting the accuracy of routine 2D anatomical linear measures?

**Material and methods:**

In a prospective randomized comparative study, patients scheduled for elective cardiac surgery underwent a comprehensive intraoperative TEE examination (Philips CX 50 with X‑72T probe). They were divided into two groups. In the routine protocol (RP) group, the TEE examination was conducted according to the standardized departmental image acquisition protocol, while in the study protocol (SP) group, multiplane views were integrated into the RP to replace the corresponding 2D views. The examinations were conducted by two experienced echocardiographers. At the end of the assigned examination protocol the timer was stopped and the missing views were obtained (2D for the X‑plane and vice versa) as well 3D datasets of the mitral valve, aortic valve and left ventricle.

Measurements of mitral and aortic annuli as well as left ventricular length from 2D and X-plane views were subsequently performed offline. Measurements obtained from a multiplanar reconstruction of a full volume (FV) 3D dataset from the same patient were used as the gold standard to compare measurements in RP and SP.

**Results:**

The examination time was significantly shorter in the SP group (SP: 481 ± 60 s; RP 595 ± 60 s; *p* < 0,001). There was no significant difference for any of the measurements using the SP and RP. The mean percentage error, although not statistically significantly different, was numerically smaller for the X‑plane than for 2D method compared to 3D except for the mitral valve annulus. Overall, X‑plane tended to show lower variability compared to 2D.

**Conclusion:**

Integrating multiplane views into a standardized comprehensive TEE image acquisition protocol reduces the examination time. The accuracy of standardized linear measurements in X‑plane mode is comparable to that of conventional 2D imaging.

## Einleitung

Eine intraoperative transösophageale Echokardiographie (TEE) sollte bei herzchirurgischen Eingriffen und Eingriffen an der thorakale Aorta sowohl nach den Empfehlungen der American Society of Echocardiography (ASE) und der Society of Cardiovascular Anesthesiologists (SCA) [3] als auch nach den Empfehlungen der Deutschen Gesellschaft für Anästhesiologie und Intensivmedizin (DGAI) [6] durchgeführt werden. Empfohlene Standardschnitte für eine umfassende TEE-Untersuchung wurden bereits 1996 veröffentlicht [4] und 2013 um multiplanare Schnittbilder und 3D-Aufnahmen ergänzt [10].

Um die Einheitlichkeit und Effizienz unserer umfassenden intraoperativen TEE-Untersuchungen zu gewährleisten, verwenden wir seit 2005 ein standardisiertes institutionelles Untersuchungsprotokoll. Die Reihenfolge der Schnittbilder wurde dabei so gewählt, dass die Bewegungen der TEE-Sonde minimiert werden. Es umfasst 22 2D-Schnittbilder und obligatorische Messungen für jedes Schnittbild. Durch die Einbindung der multiplanaren Schnittbilder (bei Philips Geräten, X‑Plane genannt) kann die Anzahl der Schnittbilder von 22 auf 16 reduziert werden.

In der vorliegenden Studie wurde der Einfluss der Integration von X‑Plane-Schnittbildern in ein umfassendes intraoperatives TEE-Untersuchungsprotokoll (Studienprotokoll) im Vergleich zum Routineprotokoll auf Effizienz und Genauigkeit von ausgewählten Messungen in der klinischen Praxis untersucht.

Der primäre Endpunkt der Studie war die benötigte Untersuchungszeit; sekundäre Endpunkte waren die Genauigkeit der Messungen der Länge des linken Ventrikels (LVL), des Mitralklappen- (MKA) und des Aortenklappenanulus (AKA) in 2D und X-Plane.

## Methodik

Nach Genehmigung durch die Ethik-Kommission (Universität Leipzig, Antragsnummer: 042/17ek) und schriftlicher Einwilligung der Patienten wurden Patienten mit einem Mindestalter von 18 Jahren, die für eine elektive herzchirurgische Operation vorgesehen waren, in diese prospektive Studie eingeschlossen. Ausschlusskriterien waren Kontraindikationen für TEE [10] und Untersuchungsabbruch.

Am Morgen des Eingriffs wurden die Patienten zu einem der beiden zertifizierten Untersucher sowie einem der beiden TEE-Untersuchungsprotokolle mittels der Methode mit versiegeltem Umschlag randomisiert: dem Routineprotokoll mit 22 2D-Schnittbildern (RP) oder dem Studienprotokoll mit 9 2D- und 7 X‑Plane-Schnittbildern (SP).

Für jedes Schnittbild waren auch spezifische Spektral- und Farbdoppleraufnahmen obligat.

Nach Anästhesieeinleitung, endotrachealer Intubation und Platzierung von arteriellen und venösen Kathetern und Einführen der X7-2t-TEE-Sonde (Fa. Philips, Handover, MA, USA) wurde vor dem Hautschnitt eine umfassende TEE-Untersuchung (CX 50, Fa. Philips, Handover, MA, USA) mit dem zugeteilten Untersuchungsprotokoll durchgeführt. Die Untersuchungszeit wurde durch eine Forschungsassistentin von der Einstellung des ersten Schnittbildes bis zur Aufzeichnung des letzten Schnittbildes in Sekunden gemessen.

Anschließend wurden bei allen Patienten zusätzlich 3D-Single-Beat-Full-Volume(FV)-Datensätze der Mitralklappe, Aortenklappe und des linken Ventrikels (LV) aufgezeichnet. Die TEE-Untersuchungen wurden auf einem eigenen Server in der Image Arena (Fa. Tomtec München, Deutschland) im DICOM-Format zur Offline-Analyse gespeichert.

Nach Abschluss der Untersuchung wurden die X‑Plane-Schnittbilder des SP bei Patienten der RP-Gruppe aufgenommen und fehlende 2D-Schnittbilder bei Patienten der SP-Gruppe. Dies diente der Ermöglichung eines Vergleichs der Messungen.

Die erforderlichen Messungen wurden vom jeweiligen Untersucher offline durchgeführt. Als Goldstandard wurden Messungen unter Verwendung der multiplanaren Rekonstruktion (MPR) der 3D-Datensätze verwendet.

Folgende Messungen wurden in die Studie aufgenommen:

- Länge des linken Ventrikels (LVL) enddiastolisch vom MK-Annulus bis zur Spitze des Ventrikels (gemessen im midösophagealen Vierkammer(4K)- und Zweikammer(2K)-Blick).
- Anteroposteriorer Durchmesser des MKA- (enddiastolisch im midösophagealen Langachsenblick) und mediolateraler Durchmesser (im midösophagealen mitralkommissuralen Blick).
- AKA (endsystolisch im midösophagealen Langachsenblick), (in der X‑Plane-Ansicht als sekundäre Ebene vom midösophagealen Kurzachsenblick auf die Aortenklappe durch Anschnitt der Mitte der AK) [14].

### Datenanalyse

Kontinuierliche Variablen wurden mit dem Shapiro-Wilk-Test auf Normalverteilung geprüft. Die Daten wurden mit dem Student-*t*-Test verglichen und sind als Mittelwert ± Standardabweichung (SD) angegeben. Kategoriale Daten wurden mit dem χ^2^-Test oder, falls angemessen, mit dem Exakten Test nach Fisher verglichen und sind als absolute Zahlen (Proportionen) dargestellt. Ein *p*-Wert < 0,05 wurde als statistisch signifikant angesehen.

Die Bland-Altman-Methode berechnet die mittlere Differenz zwischen 2 Messmethoden („Bias“) sowie die 95 %-Übereinstimmungsgrenzen (Limits of Agreement, LOA) als mittlere Differenz ± 2 Standardabweichungen (SD). Je kleiner der Abstand zwischen diesen beiden Grenzen ist, desto besser ist die Übereinstimmung der Messungen. Ein mittlerer prozentualer Fehler (Percentage Error, PE) < 30 % gilt ebenfalls als Hinweis auf eine ausreichende Übereinstimmung zwischen den Messmethoden [5, 8].

Die Korrelation wurde mittels Pearson-Korrelationskoeffizienten (r) bestimmt. Zum Vergleich der mittleren Differenzen (MOD) zwischen 2D vs. 3D sowie X‑Plane vs. 3D wurde für jedes Datenset ein gepaarter *t*-Test angewendet. Um die Korrelationskoeffizienten (r) zwischen den Methodenpaaren zu vergleichen, kam die Fisher-z-Transformation zum Einsatz. Abschließend wurde ein gepaarter *t*-Test verwendet, um die Bias aller Vergleiche (2D vs. 3D) und (X-Plane vs. 3D) zu vergleichen.

Für die statistische Analyse wurde StatsDirect (Version 3.3.5, Fa. StatsDirect Ltd, Cheshire, UK) verwendet.

Die Schätzung der Stichprobengröße basierte auf einer erwarteten Differenz in der Untersuchungszeit unter Verwendung der beiden Protokolle von 2 min, mit einem α‑Fehler von 0,05 und einer Power von 95 %. Dies ergab eine Mindestanzahl von 26 Patienten/Protokoll. Unter Annahme einer Abbruchquote von 10 % sollten mindestens 29 Patienten/Protokoll eingeschlossen werden.

## Ergebnisse

In die Studie wurden 64 Patienten eingeschlossen. Bezüglich Alter, Geschlecht und durchgeführter Operation gab es zwischen den beiden Gruppen keine signifikanten Unterschiede (Tab. 1).

**Tab. 1:** Patientenmerkmale

		Gesamt *n* = 64	Routineprotokoll *n* = 32	Studienprotokoll *n* = 32	*p*-Wert
Alter (Jahre)		63 ± 12	63 ± 11	62 ± 13	0,57
Geschlecht	W	19 (29,7 %)	12 (18,7 %)	7 (11 %)	0,36
	M	45 (70 %)	20 (31 %)	25 (39 %)	0,55
Operation	Bypass-Operation	22 (34 %)	12 (37 %)	10 (31 %)	0,83
	Einzelklappenoperation	24 (37 %)	11 (34 %)	13 (40 %)	0,84
	Zweifachklappenoperation	04 (6 %)	03 (9 %)	01 (3 %)	0,63
	Aortenchirurgie	05 (8 %)	02 (6 %)	03 (9 %)	> 1
	Bypass + Einzelklappenoperation	7 (11 %)	3 (9 %)	4 (12 %)	0,73
	Andere	2 (3 %)	1 (3 %)	1 (3 %)	> 1
Untersucher	A	32 (50 %)	16 (50 %)	16 (50 %)	–
	B	32 (50 %)	16 (50 %)	16 (50 %)	–

Werte als Mittelwert ± Standardabweichung oder als Anzahl und Prozent

*W* Weiblich; *M* Männlich

Die Verwendung des Studienprotokolls führte zu einer Verkürzung der Untersuchungszeit um 115 s (Durchschnittszeit: SP: 481 ± 60 s vs. RP 595 ± 60 s) (*p* < 0,001).

Von den insgesamt 64 Patienten standen 62 vollständige und qualitativ hochwertige Datensätze zur Verfügung, die für die Analyse herangezogen werden konnten. In einem Fall aus der RP-Gruppe und in einem Fall aus der SP-Gruppe waren die Datensätze entweder unvollständig oder die Qualität reichte nicht aus, um präzise Messungen durchzuführen.

**Abb. 1:**
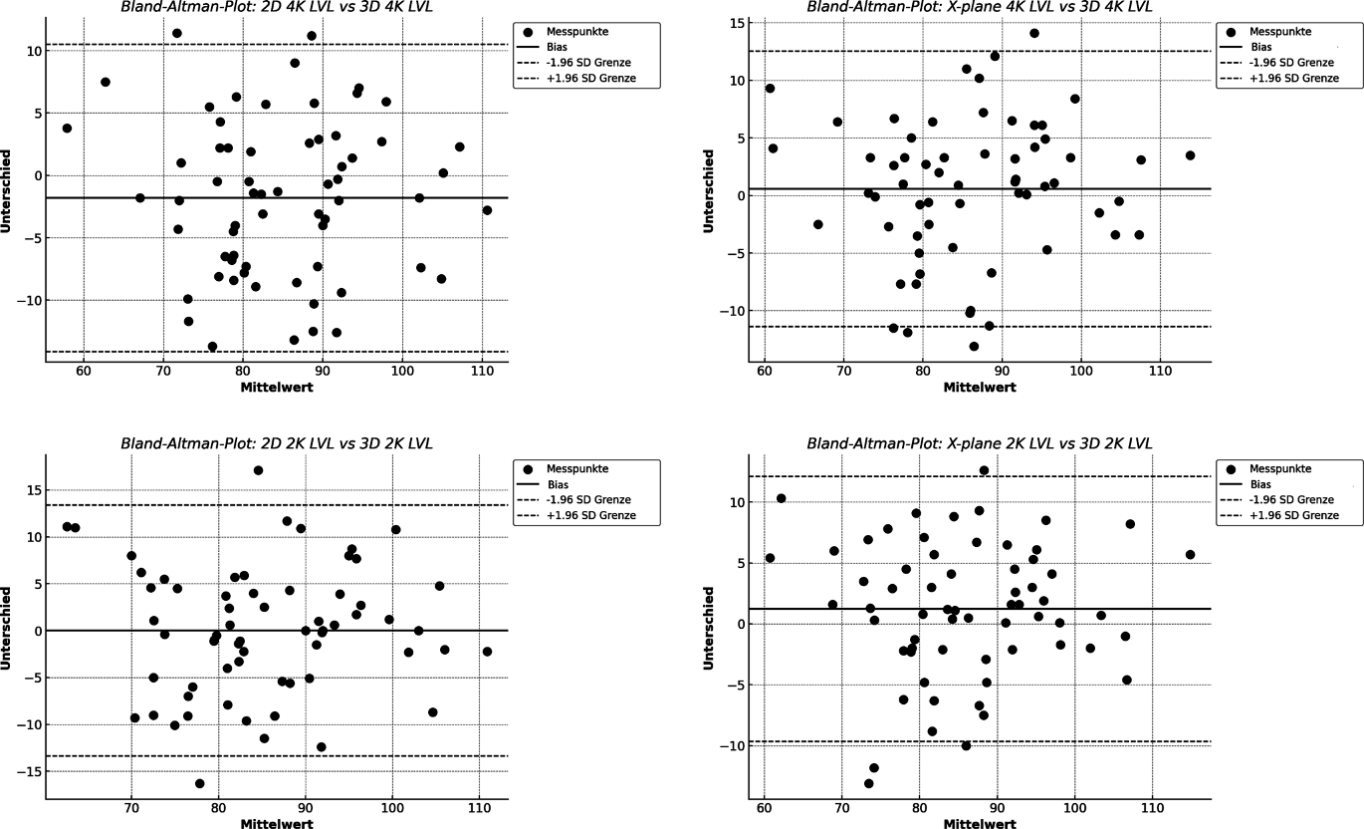
Bland-Altman-Diagramm, Länge des linken Ventrikels (*LVL*). *4K* midösophagealer Vierkammerblick; *2K* midösophagealer Zweikammerblick

Der Bland-Altman-Vergleich der 2D- und X-Plane-Messungen mit der 3D-Referenz zeigte keinen signifikanten Unterschied zwischen dem Routine- und dem Studienprotokoll. Im Vergleich zur 2D-Methode war der mittlere prozentuale Fehler bei der X‑Plane-Methode geringer, außer bei der Messung des MKA in der ME-MC-Ansicht. Bei allen Vergleichen variierte die mittlere Differenz (Bias) zwischen 2D und X-Plane. Insgesamt zeigte X‑Plane tendenziell eine geringere Variabilität im Vergleich zu 2D, was sich in einer niedrigeren Standardabweichung widerspiegelte. In den meisten Fällen wies X‑Plane engere limits of agreement auf und zeigte über alle Vergleiche hinweg eine konsistent stärkere Korrelation mit der 3D-Referenzmethode (Tab. 2; Abb. 1 und 2).

**Tab. 2:** Vergleich der Messungen (mm) im 2D- sowie im X‑Plane-Modus mit 3D-FV

		Bias	SDD	Unterer Wert der LOA	Oberer Wert der LOA	Prozentsatz der Punkte außerhalb der LOA (%)	*r*	*p*‑Wert für Korrelation 2D und X‑Plane gegen 3D	MPF gegen 3D (%)	Fischer z‑transformation für 2D gegen X‑Plane	Gepaarte *t*-Test für 2D gegen X‑Plane Bias
4K LVL	2D	−1,8	6,28	−14,11	10,51	3,13	0,83	< 0,001	6,32	−0,07	0,95
	X‑Plane	0,57	6,10	−11,39	12,54	4,68	0,85	< 0,001	5,74		
2K LVL	2D	0,03	6,81	−13,34	13,39	3,13	0,81	< 0,001	6,54	−0,21	0,84
	X‑Plane	1,23	5,54	−9,64	12,11	6,25	0,87	< 0,001	5,54		
MC MKA	2D	0,31	3,58	−6,72	7,34	4,68	0,79	< 0,001	8,13	−0,09	0,93
	X‑Plane	0,72	3,20	−5,55	6,99	1,56	0,82	< 0,001	8,23		
LAX MKA	2D	0,73	3,76	−6,64	8,10	6,25	0,75	< 0,001	9,23	−0,22	0,83
	X‑Plane	1,08	2,84	−4,49	6,66	6,25	0,83	< 0,001	7,54		
LAX AKA	2D	−0,37	1,5	−3,49	2,73	3,13	0,86	< 0,001	5,09	−0,3	0,77
	X‑Plane	0,06	1,22	−2,33	2,45	6,25	0,92	< 0,001	3,43		

*LVL* linksventrikuläre Länge; *4K* midösophagealer Vierkammerblick; *2K* midösophagealer Zweikammerblick; *MKA* Mitralklappenanulus; *MC* midösophagealer Mitralkommissuralblick; *AKA* Aortenklappenanulus; *LAX* midösophagealer Langachsenblick; *CI* Konfidenzintervall; *SDD* Standardabweichung der Differenzen; *LOA* Limits of Agreement; *r* Korrelationskoeffizient nach Pearson; *MPF* mittlerer prozentualer Fehler

**Abb. 2:**
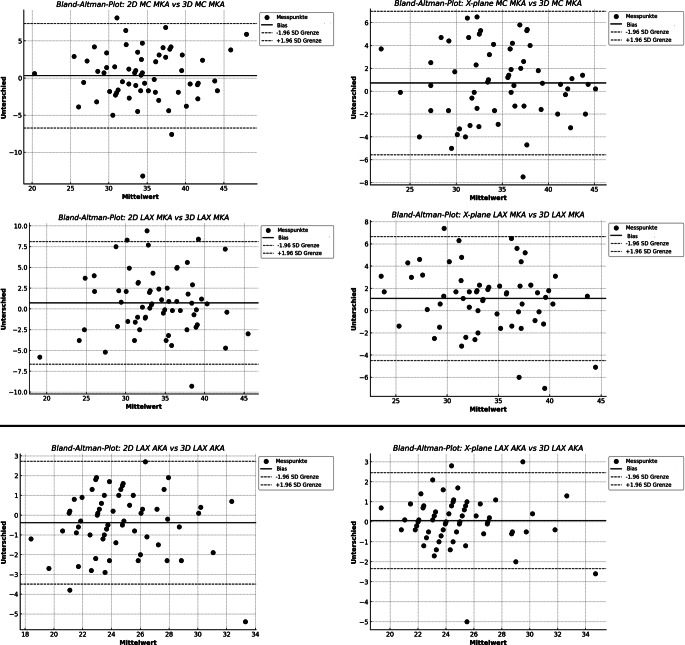
Bland-Altman-Diagramm, Mitral- und Aortenklappenanulus. *MKA* Mitralklappenanulus; *AKA* Aortenklappenanulus; *MC* midösophagealer mitralkommissuraler Blick; *LAX* midösophagealer Langachsenblick

Der Korrelationsvergleich unter Anwendung der Fisher-z-Transformation zeigt, dass zwischen den beiden Methoden über alle Datensätze hinweg kein signifikanter Unterschied in der Stärke der Korrelation besteht. Der *t*-Test zeigt, dass die mittleren Differenzen (MOD) zwischen (2D vs. 3D) und (X-Plane vs. 3D) nicht signifikant unterschiedlich sind (*p* = 0,0699).

Die Bildrate betrug in fast allen Schnittbildern der Messungen in 2D 46 Hz und in X‑Plane 23 Hz (Tab. 3).

**Tab. 3:** Durchschnittliche Bildrate (Bilder/s) der untersuchten Standardschnitte

	2D	X‑Plane	3D
LVL 4K	46	23	11
LVL 2K	46	23	11
MKA MC	46	23	10
MKA LAX	46	23	10
AKA LAX	46	23	10

*LVL* linksventrikuläre Länge; *4K* midösophagealer Vierkammerblick; *2K* midösophagealer Zweikammerblick; *MKA* Mitralklappenanulus; *MC* midösophagealer Mitralkommissuralblick; *AKA* Aortenklappenanulus; *LAX* midösophagealer Langachsenblick

## Diskussion

In dieser Studie konnte gezeigt werden, dass ein Untersuchungsprotokoll, welches multiplanare Schnittbilder integriert, zu einer signifikanten Verkürzung der Untersuchungsdauer führt, ohne die Qualität zu beeinträchtigen. Die beobachtete Reduktion der Untersuchungszeit durch die Verwendung des Studienprotokolls betrug fast 2 min (114,6 s). Dies entspricht etwa 20 % der Zeit, die in dieser Kohorte für die Durchführung einer umfassenden Untersuchung unter Verwendung des Standardprotokolls benötigt wurde. Eine Umfrage an deutschen kardioanästhesiologischen Abteilungen ergab, dass die verfügbare Zeit bei 48 % der Kliniken der limitierende Faktor für die Durchführung einer umfassenden TEE-Untersuchung war [9]. In diesem Kontext könnte ein standardisiertes TEE-Untersuchungsprotokoll einschließlich X‑Plane-Schnittbildern dazu beitragen, die Durchführung einer umfassenden TEE-Untersuchung gemäß den Leitlinien der ASE und SCA [10, 13] in kürzerer Untersuchungszeit zu gewährleisten.

Wir verwendeten 3D-TEE-Datensätze als Goldstandard, da die Schnittebenen mittels MPR korrigiert werden können und somit speziell im Vierkammer- und Zweikammerblick ein „Foreshortening“ des LV vermieden werden kann [14]. Verschiedene Studien haben gezeigt, dass Messungen mittels 3D-Echokardiographie eine bessere Korrelation mit Computertomographiemessungen aufweisen als 2D-Echokardiographie [2, 7, 11, 12, 15]. Korrekte Messungen sind zum einen davon abhängig, dass die Schnittebene korrekt eingestellt ist, und zum anderen, dass die zeitliche Auflösung hoch genug ist.

In der klinischen Anwendung deutet ein Verhältnis der Differenz der LVL zwischen midösophagealem 2K- und 4K-Blick von mehr als 10 % auf eine perspektivische Verkürzung hin, die zu einem signifikanten Fehler bei der Berechnung der linksventrikulären Ejektionsfraktion nach der „method of the disks“ führen kann [14]. In unserer Studie gab es keinen signifikanten Unterschied zwischen den 2D- und den X‑Plane-Messungen. Im Gegensatz dazu zeigte die LVL-Messung mittels 2D-TEE- und 3D-Datensatz in einer anderen Studie jedoch eine signifikante Abweichung zwischen den Methoden [1]. Ein wesentlicher Unterschied im Studiendesign beider Studie war, dass in der vorliegenden prospektiven Studie nur 2 erfahrene Untersucher die Bilder generierten, während in der zitierten Studie auch weniger erfahrene Untersucher beteiligt waren.

Bei der MKA- und AKA-Messung zeigt unsere Studie eine gute Korrelation der beiden Methoden mit 3D und keinen signifikanten Unterschied zwischen den Methoden.

Insgesamt zeigt X‑Plane numerisch eine bessere Übereinstimmung mit der 3D-Referenzmethode, was sich in einem geringeren mittleren prozentualen Fehler gegen 3D widerspiegelt. Ein Unterschied < 30 % des prozentualen Fehlers zweier unterschiedlicher Messverfahren spricht für eine ausreichende Übereinstimmung. In dieser Studie lag der prozentuale Fehler < 10 % (3,43–9,23). Somit sind aus klinischer Sicht beide Verfahren als gleichwertig anzusehen. Da sämtliche Analysen von erfahrenen Untersuchern durchgeführt wurden, ist anzunehmen, dass bei weniger erfahrenen Anwendern größere Abweichungen – insbesondere bei 2D-Messungen – auftreten können. Bei der Bildqualität sind prinzipiell sowohl die räumliche als auch die zeitliche Auflösung zu berücksichtigen. Gerade bei der zeitlichen Auflösung kommt es bei Verwendung des X‑Plane-Modus und der Single-Beat Acquisition im 3D-FV-Datensatz zu einer Verringerung der Bildrate und somit zu einer geringen zeitlichen Auflösung. In unserer Studie halbierte sich die Bildrate von 2D zu X‑Plane. Wir konnten aber zeigen, dass dies bezüglich der Messungen zu keinen signifikanten Fehlmessungen führt, da sie innerhalb der Standardabweichung der 3D-Messungen lagen. Dies mag daran liegen, dass die Bildrate bei der X‑Plane-Darstellung immer noch über 20 Hz lag (Tab. 3). Die niedrigere Bildrate im 3D-FV-Datensatz von 10 Hz kann anscheinend vernachlässigt werden.

### Limitationen

Beide Untersucher waren sehr erfahren (mehr als 100 TEE-Untersuchungen/Jahr und mehr als 10 Jahre Erfahrung). Dies begrenzt die allgemeine Übertragbarkeit unserer Ergebnisse. Es ist jedoch anzunehmen, dass der Zeitvorteil bei unerfahreneren Untersuchern noch größer ausfallen würde. Beide Untersucher waren darüber hinaus während der echokardiographischen Untersuchung nicht in die Versorgung der untersuchten Patienten involviert. Dies ist in der klinischen Routine in den meisten Kliniken eher nicht zu gewährleisten. Der primäre Endpunkt der Studie war die Dauer der reinen Untersuchungszeit. Die aktive Interpretation der Bilddaten sowie der Zeitaufwand für die Durchführung der Messungen wurden nicht in die Analyse einbezogen. Diese Abgrenzung erfolgte, da zunächst die für die Messungen erforderlichen Bildaufnahmen generiert werden müssen. Die eigentliche Auswertung der Messdaten erfolgt – entsprechend der klinischen Routine – üblicherweise offline und beeinflusst daher die Dauer der Bildakquisition nicht.

Des Weiteren war die Forschungsassistentin weder gegenüber dem Untersucher, der die TEE-Untersuchung durchführte, noch gegenüber dem verwendeten Protokoll verblindet.

## Schlussfolgerung

Unsere Studie zeigt, dass der X‑Plane-Modus die Dauer einer vollständigen TEE-Untersuchung deutlich verkürzt und bei standardisierten linearen Messungen eine mit der 2D-Bildgebung vergleichbare Genauigkeit bietet. Somit könnten X‑Plane-Schnittbilder ein wertvolles Werkzeug sein, eine umfassende TEE-Untersuchung, auch bei begrenzter Untersuchungszeit, zu gewährleisten.

## Fazit für die Praxis

- Der X‑Plane-Modus ermöglicht die gleichzeitige Darstellung zweier senkrecht zueinanderstehender Schnittebenen während der intraoperativen TEE.
- Die Integration des X‑Plane-Modus in ein intraoperatives TEE-Protokoll verkürzt die Dauer einer vollständigen TEE-Untersuchung signifikant.
- Die Genauigkeit standardisierter linearer Messungen im X‑Plane-Modus ist mit derjenigen der konventionellen 2D-Bildgebung vergleichbar.

## Data Availability

Die in dieser Studie erhobenen Datensätze können auf begründete Anfrage beim Korrespondenzautor angefordert werden.
